# Simultaneous Improvement of Yield Strength and Ductility at Cryogenic Temperature by Gradient Structure in 304 Stainless Steel

**DOI:** 10.3390/nano11071856

**Published:** 2021-07-19

**Authors:** Shuang Qin, Muxin Yang, Fuping Yuan, Xiaolei Wu

**Affiliations:** 1 State Key Laboratory of Nonlinear Mechanics, Institute of Mechanics, CAS, 15 Beisihuan West Road, Beijing 100190, China; qinshuang@imech.ac.cn (S.Q.); xlwu@imech.ac.cn (X.W.); 2 School of Engineering Science, University of Chinese Academy of Sciences, 19A Yuquan Road, Beijing 100049, China

**Keywords:** gradient structure, ductility, strain hardening, martensitic transformation, hetero-de-formation-induced hardening

## Abstract

The tensile properties and the corresponding deformation mechanism of the graded 304 stainless steel (ss) at both room and cryogenic temperatures were investigated and compared with those of the coarse-grained (CGed) 304 ss. Gradient structures were found to have excellent synergy of strength and ductility at room temperature, and both the yield strength and the uniform elongation were found to be simultaneously improved at cryogenic temperature in the gradient structures, as compared to those for the CG sample. The hetero-deformation-induced (HDI) hardening was found to play a more important role in the gradient structures as compared to the CG sample and be more obvious at cryogenic temperature as compared to that at room temperature. The central layer in the gradient structures provides stronger strain hardening during tensile deformation at both temperatures, due to more volume fraction of martensitic transformation. The volume fraction of martensitic transformation in the gradient structures was found to be much higher at cryogenic temperature, resulting in a much stronger strain hardening at cryogenic temperature. The amount of martensitic transformation at the central layer of the gradient structures is observed to be even higher than that for the CG sample at cryogenic temperature, which is one of the origins for the simultaneous improvement of strength and ductility by the gradient structures at cryogenic temperature.

## 1. Introduction

Metals and alloys with both high strength and large ductility are always desirable in the applications of industry [1,2]. High strength can be achieved in metals and alloys by well-known strategies, such as cold working and grain refinement. However, the elevation in strength by such strategies is usually accompanied by the sacrifice of ductility [3,4,5,6]. The diminished ductility for high-strength metals and alloys can often be attributed to the low strain hardening rate [5,6]. Recently, several novel strategies for achieving excellent tensile properties have been proposed, such as gradient structures [7,8], heterogeneous lamella structure [9,10,11,12], heterogeneous grain structure [13,14], nanodomained structure [15], nano-twins [16,17] and nano-precipitates [18,19]. Among them, gradient structures have attracted extensive research interests in the last decade for their potential in achieving extraordinary synergy of strength and ductility by tailoring their microstructures [17,20,21,22].

Once spatial gradients in the local chemical composition, phase constituents and/or microstructural characteristics are introduced on the surface of metallic materials, the mechanical properties and functional performance of these metals and alloys are improved [23,24,25,26]. To optimize the mechanical properties and performance, chemical and/or structural gradients have been carefully designed in engineering materials [8,27,28]. The gradient structures often consist of gradient layers with varying grain size or volume fraction of twins or texture or dislocation density along the depth [27,29]. The high ductility in gradient structures can be attributed to the extra strain hardening due to the change of stress states and the generation of strain gradient [8,21], which can promote geometrically necessary dislocations (GNDs) and hetero-deformation-induced (HDI) hardening [30,31,32]. The synergetic strengthening/hardening is due to the macroscopic strength gradient and mechanical incompatibility among different layers; thus, the stress/strain partitioning among different layers can generate a high density of GNDs for excellent tensile properties [30,31,32,33,34].

The strategy of gradient structure has been applied to the 304 austenite stainless steel (ss) for producing both a high strength and large ductility in previous research [28,35]. The 304 ss can be considered as the model material for transformation-induced-plasticity (TRIP) effect or twinning-induced-plasticity (TWIP) effect during tensile deformation. In these studies [28,35,36,37], the mechanical properties, the plastic deformation and strain hardening mechanisms for the graded 304 ss were systematically investigated. It has been revealed that martensitic transformation is triggered successively (from layer to layer) with increasing applied tensile strain. It was found that the gradient structures can prolong the TRIP effect to large tensile strains. Thus, the excellent combination of strength and ductility in the graded 304 ss can be attributed to the dynamic strain partitioning and the successively triggered TRIP effect among different layers [28].

Furthermore, 304 ss can be considered an ideal candidate material for cryogenic applications, due to its extraordinary mechanical properties at cryogenic temperature [38,39,40,41]. Since the stacking fault energy (SFE) is generally lower at cryogenic temperature, the TRIP effect or TWIP effect usually becomes more dominant with decreasing temperature in 304 ss, resulting in stronger strain hardening and better tensile properties [38,39,42]. Meanwhile, 304 ss with coarse grains (CGs) has a low yield strength, limiting its applications. The graded 304 ss can have the potential for obtaining both high strength and large ductility, while the mechanical properties and the deformation mechanisms at cryogenic temperature for the graded 304 ss were still not explored so far. Therefore, there are two open questions for the gradient structures: whether gradient structure effect works at cryogenic temperature, and how different gradient structures might behave as compared with that at room temperature; both of them are the focus and the concerned novelties of this work. In this regard, gradient structures were produced in 304 ss, and then the tensile properties and the deformation mechanisms at both room and cryogenic temperatures for the graded 304 ss and the CGed 304 ss were revealed and compared in the present study.

## 2. Materials and Methods

In the present study, the chemical composition of the AISI 304 ss is 0.04 C, 0.49 Si, 1.65 Mn, 7.8 Ni, 16.8 Cr, 0.37 Mo and the balance of Fe (all in weight%). The 304 ss with CGs was annealed and austenitized at 1273 K for 15 min. These annealed samples are referred as CG samples. Then the as-annealed disks with a diameter of 100 mm and a thickness of 1.5 mm were processed by the surface mechanical attrition treatment (SMAT) technique to produce the gradient structures. During the SMAT process, spherical steel balls with 3 mm diameter were accelerated by the high frequency system (20 kHz) to impact both sides of the disks for 10 min. After the SMAT process, the SMATed disks were hot-rolled at 823 K, with a thickness reduction of 33%. The hot-rolled samples are referred to as GS1 samples. Then the hot-rolled samples were annealed at 973 K for 2 min, and these short-time annealed samples are referred to as GS2 samples.

The dimensions of gauge section of the dog-bone plate specimens for tensile testing are 1.0 × 3.0 × 15 mm^3^. Using an Instron 5565 testing machine (Instron Inc., Norwood, MA, USA), the quasi-static uniaxial tensile tests and load–unload–reload (LUR) tests were conducted at a strain rate of 5 × 10^−4^/s at both room and cryogenic temperatures under displacement control. For LUR tests, the samples were first elongated to a designated strain at the strain rate of 5 × 10^−4^/s, and then the samples were unloaded to 20 N by the stress-control mode (unloading rate of 200 N/min), followed by reloading at the strain rate of 5 × 10^−4^/s. An extensometer (NCS Testing Technology Co., Ltd., Beijing, China) was utilized to accurately measure and control the displacement during the tensile tests and LUR tests. The distributions of micro-hardness along the depth for the gradient structures before and after tensile testing were measured on the polished sample surfaces, using a Vickers diamond indenter under a load of 10 g for 15 s dwell time. Ten groups of measurements for each point were obtained, the average value was taken and the error bar was also provided.

Before and after tensile testing, electron back-scattered diffraction (EBSD, Oxford Instruments PLC. Oxford, UK) was used to characterize the microstructures. The sample preparation for EBSD observations can be found in our previous paper [43]. A minimum scanning step of 100 nm was used for EBSD acquisition. Kernel average misorientation (KAM) was calculated against the first nearest neighbor ignoring the misorientation larger than 3 degrees [44,45].

## 3. Results and Discussions

**Figure 1:**
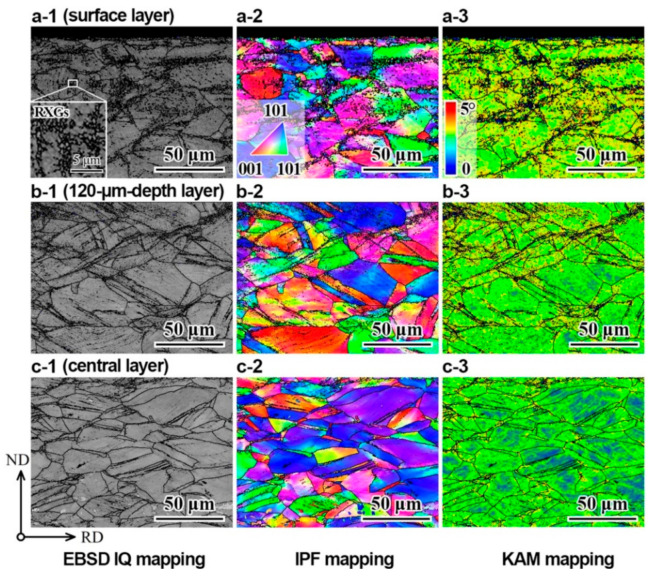
EBSD images of the GS2 sample prior to tensile testing: (**a1**–**a3**) IQ, IPF and KAM mappings for the surface layer; (**b1**–**b3**) IQ, IPF and KAM mappings for the layer with a depth of 120 µm; (**c1**–**c3**) IQ, IPF and KAM mappings for the central layer.

The microstructures at various depths for the GS2 sample are characterized by EBSD and are shown in Figure 1. The microstructures at the surface, at the depth of 120 µm and at the center are displayed in Figure 1a(1–3), Figure 1b(1–3) and Figure 1c(1–3), respectively. In these figures, Figure 1a(1–3) shows the image quality (IQ) maps, Figure 1b(1–3) displays the inverse pole figure (IPF) images and Figure 1c(1–3) shows the KAM distribution images.

According to the phase maps at various depths for the GS2 sample, most of the grains are austenite (nearly 100% austenite) at all depths for the GS2 sample, which means that the grains are fully austenited by the short-time annealing. As clearly indicated in the inset of Figure 1(a1), that some small recrystallized grains (RXGs) are formed in the CGs after the SMAT and the followed annealing processes. The size of these small grains is less than 1 µm; thus, they can be considered ultrafine grains (UFGs). It is generally thought that the surface layer with more stored deformation energy during the SMAT is easier to form RXGs during the subsequent short-time annealing [46,47], which is consistent with the observations in Figure 1.

The KAM value generally can be reflected to GNDs induced by plastic deformation [19]. In general, the GND density can be calculated by using a method based on the strain gradient theory, which was proposed by Gao and Kubin [48,49]: $\rho_{GND}=2\theta /lb$, where $\rho_{GND}$ is the GND density at local points, θ represents the misorientation at local points, l is the unit length (100 nm in this work) for the local points and b is the Burger’s vector for the materials (0.253 nm for 304 ss). The average KAM value is found to decrease from the surface layer to the center.

**Figure 2:**
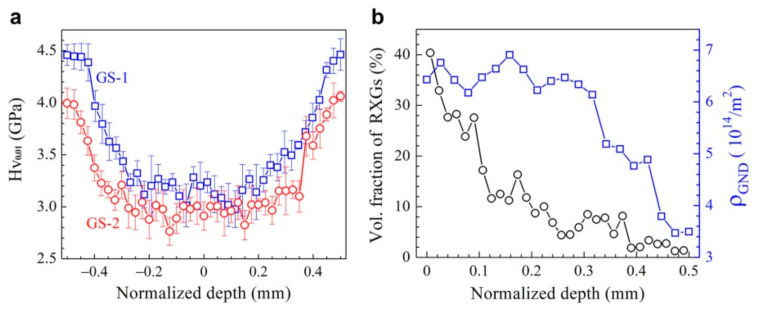
(**a**) The micro-hardness distributions along the depth for the GS1 and GS2 samples. (**b**) The relative area ratio of RXGs (UFGs) and the average GND density ($\rho_{GND}$) as a function of normalized depth for the GS2 sample.

The micro-hardness distributions along the depth for the GS1 and GS2 samples are shown in Figure 2a. The average micro-hardness for the CG sample is about 2 GPa. As indicated in Figure 2a, both the GS1 and GS2 samples show strong hardness gradient from surface to the depth of about 300 µm at both sides. A hardness plateau is also observed for the central layer (about 400 µm thick) for both samples, while this central layer is also hardened during SMAT process, since the plateau hardness of this center layer is much higher than that of the CG samples (2 GPa). Thus, those two gradient samples (GS1 and GS2) can be considered as a central layer with a thickness of about 400 µm sandwiched by two gradient layers with a thickness of about 300 µm. It is also interesting to note that the hardness for the GS1 samples is higher than that for the GS2 sample at each depth. The relative area ratio of RXGs (UFGs) and the average GND density ($\rho_{GND}$) are plotted as a function of normalized depth for the GS2 sample in Figure 2b, which both show obvious gradients along the depth. Thus, the hardness gradient in Figure 2a should be induced by the gradients of the relative area ratio of RXGs (UFGs) and the average GND density along the depth.

**Figure 3:**
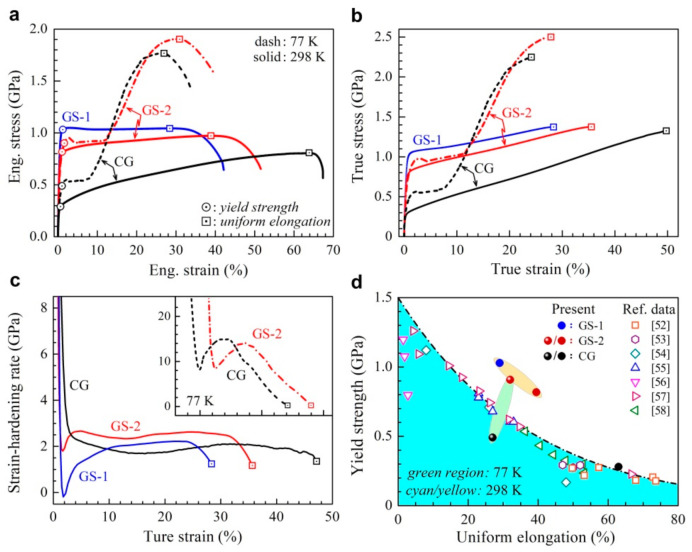
The tensile properties of CG, GS1 and GS2 samples at both room and cryogenic temperatures. (**a**) The engineering stress–strain curve. (**b**) The true stress–strain curves. (**c**) The hardening rate–true strain curves. (**d**) The yield strength as a function of the uniform elongation for our results, along with the data from previous research for 304 ss [52,53,54,55,56,57,58].

The tensile properties of CG, GS1 and GS2 samples at both room and cryogenic temperatures are shown in Figure 3. The engineering stress–strain curves are displayed in Figure 3a, while the hardening rate and the true stress as a function of true strain are plotted in Figure 3b,c, respectively. In Figure 3a, the points for yielding and ultimate strength are marked by circles and squares, respectively. The yield strength and the uniform elongation of the CG sample at room temperature are about 290 MPa and 64%, respectively. Meanwhile the yield strength at room temperature is elevated to about 817 and 1030 MPa for the GS2 and GS1 samples, and moderate ductility is still maintained for these two samples (with uniform elongations of about 39% and 29%). These observations indicate that excellent synergy of strength and ductility can be obtained by gradient structures. For the CG sample, the hardening rate at room temperature decreases monotonically with increasing applied tensile strain. However, the hardening rates at room temperature for the two graded samples drop rapidly first, followed by an upturn to the maximum points, and drop again slowly until necking. Such a transient hardening behavior can be attributed to the HDI hardening by the back stress [19]. This transient upturn hardening behavior is induced by the heterogeneous elastoplastic deformation among various layers at various depths, resulting in better tensile properties.

For the CG and the GS2 samples, the yield strength is observed to be higher and the uniform elongation is found to be lower at cryogenic temperature, compared to those at room temperature. However, the tensile curves at cryogenic temperature show a two-stage strain hardening behavior for both the CG and the GS2 samples, namely an initial plateau stage followed by a second hardening stage. Strong strain hardening is observed in the second hardening stage, which generally can be attributed to the martensitic transformation after a critical tensile strain [50,51]. It is also interesting to note that both the yield strength and the uniform elongation are higher at cryogenic temperature for the GS2 sample, as compared to the CG sample. Interestingly, both the CG sample and the GS2 sample show the upturn hardening behavior at cryogenic temperature, which can be attributed to the two-stage hardening behavior. Then the yield strength is plotted as a function of the uniform elongation for our results in Figure 3d, along with the data from previous research for 304 ss [52,53,54,55,56,57,58]. It is indicated that the graded 304 ss shows an outstanding combination of the strength and the uniform elongation, compared to the homogeneous structures. Moreover, a simultaneously improvement in the strength and the uniform elongation is found at cryogenic temperature for the GS2 sample, as compared to the CG sample.

**Figure 4:**
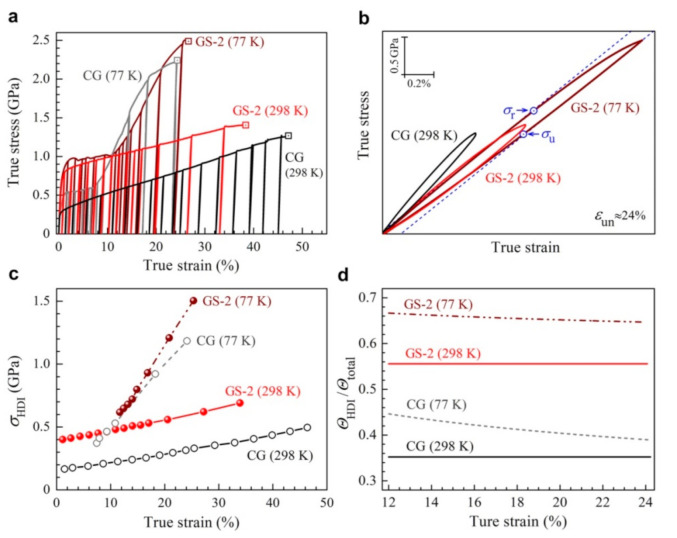
HDI hardening in the CG and GS2 samples at both room and cryogenic temperatures. (**a**) The true stress–strain curves for LUR tests. (**b**) The typical hysteresis loops at an unloading strain of 24%. (**c**) The curves of the HDI stress as a function of the true strain. (**d**) The curves of the $\Theta_{\mathrm{HDI}}/\Theta_{total}$ as a function of the true strain.

According to the previous papers [30,31,32,59], excellent tensile properties can be achieved by heterogeneous structures due to the HDI hardening, which can be attributed to the back stress that arises from plastic deformation incompatibility between hard and soft domains. In the gradient structures, the various layers at different depths have various hardness and strain hardening ability; thus, these layers can be considered as hard and soft domains, and HDI hardening should play an important role during tensile loading for the gradient structures [31,60]. In order to illustrate and compare the HDI hardening effects on the tensile properties for the CG and GS2 samples at both room and cryogenic temperatures, the corresponding LUR tests were conducted, and the true stress–strain curves for LUR tests are shown in Figure 4a. The typical hysteresis loops for the CG and GS2 samples at both room and cryogenic temperatures at an unloading strain of 24% are displayed in Figure 4b. Based on the method proposed in our previous paper [61], the back stress can be estimated by the average value of the unloading yield stress and the reloading yield stress ($\sigma_{\mathrm{back}}=\left(\sigma_{u}+\sigma_{r}\right)/2$) from the hysteresis loops of LUR tests, as indicated in the Figure 4b. The HDI hardening based on the back stress is shown in Figure 4c for the CG and GS2 samples, at both room and cryogenic temperatures. It is clearly indicated that the HDI hardening rate (the slopes for the curves in Figure 4c) is much higher at cryogenic temperature than that at room temperature.

In general, the ratio between the HDI hardening rate and the total hardening rate ($\Theta_{\mathrm{HDI}}/\Theta_{total}$) could be considered as the contribution of HDI hardening [21] to the overall strain hardening; thus, $\Theta_{\mathrm{HDI}}/\Theta_{total}$ is plotted as a function of applied tensile strain for the CG sample and the GS2 sample at both room and cryogenic temperatures in Figure 4d. It is clearly indicated that the values of $\Theta_{\mathrm{HDI}}/\Theta_{total}$ for the GS sample are higher than those for the CG sample at both room and cryogenic temperatures, resulting in better tensile properties for the GS2 sample. It is also interesting to note that the values of $\Theta_{\mathrm{HDI}}/\Theta_{total}$ at cryogenic temperature are higher than those at room temperature for both samples, resulting in better overall strain hardening at cryogenic temperature. These observations indicate that the HDI hardening plays a more important role in the GS sample than in the CG sample and plays a more important role at cryogenic temperature than at room temperature.

**Figure 5:**
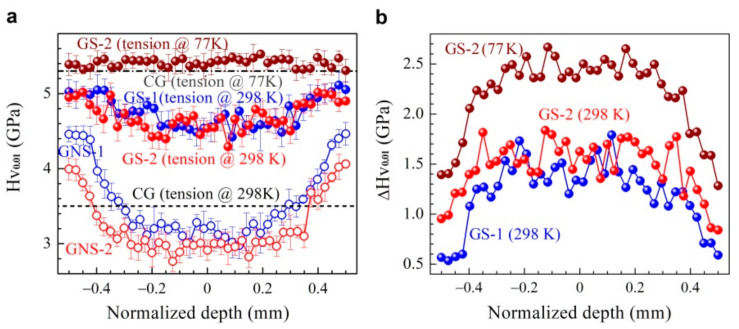
(**a**) The micro-hardness distributions along the depth for the GS1 and GS2 samples, prior to tensile testing (GS1 and GS2) and after tensile testing at both room temperature (GS1 and GS2) and cryogenic temperature (GS2), in which the average hardness in the CG sample is marked as a black dash line and a dash-dot line after tensile testing at both room and cryogenic temperatures. (**b**) The hardness increment distributions along the depth for the GS1 and GS2 samples after tensile testing at both room temperature (GS1 and GS2) and cryogenic temperature (GS2).

The hardness increment after tensile loading can generally be considered as an indicator of strain hardening ability. Thus, the micro-hardness distributions along the depth for the GS1 and the GS2 samples after tensile testing at both room temperature (GS1 and GS2) and cryogenic temperature (GS2) were measured and are displayed in Figure 5a. For a comparison, the average hardness in the CG sample after tensile testing at both room and cryogenic temperatures was also measured and is marked as a black dashed line and a dash–dot line in Figure 5a, respectively. It is clear that the micro-hardness at various depths for the GS1 and GS2 samples after testing at room temperature is much higher than the average hardness for the CG sample at the same applied tensile strain after testing at room temperature, indicating that the gradient structures can promote extra strain hardening for better tensile properties. Then the hardness increment distributions along the depth for the GS1 and GS2 samples after tensile testing at both room temperature (GS1 and GS2) and cryogenic temperature (GS2) were obtained, and they are displayed in Figure 5b. In the gradient structures, the central layer provides stronger strain hardening during tensile deformation, while the surface layer also provides a moderate strain hardening due to the gradient effect. Moreover, the hardness increment at each depth is much higher after testing at cryogenic temperature than that after testing at room temperature, indicating much stronger strain hardening at cryogenic temperature, which is consistent with the results in Figure 3.

**Figure 6:**
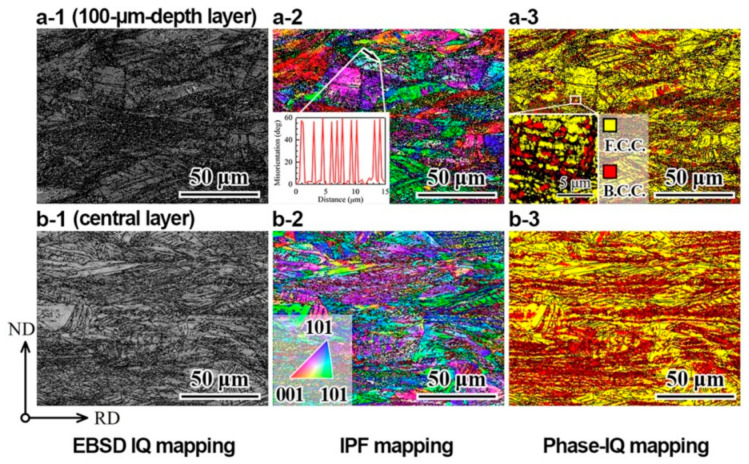
EBSD images of the GS2 sample after tensile testing at room temperature: (**a1**–**a3**) IQ, IPF and phase mappings for the layer with a depth of 100 µm; (**b1**–**b3**) IQ, IPF and phase mappings for the central layer.

**Figure 7:**
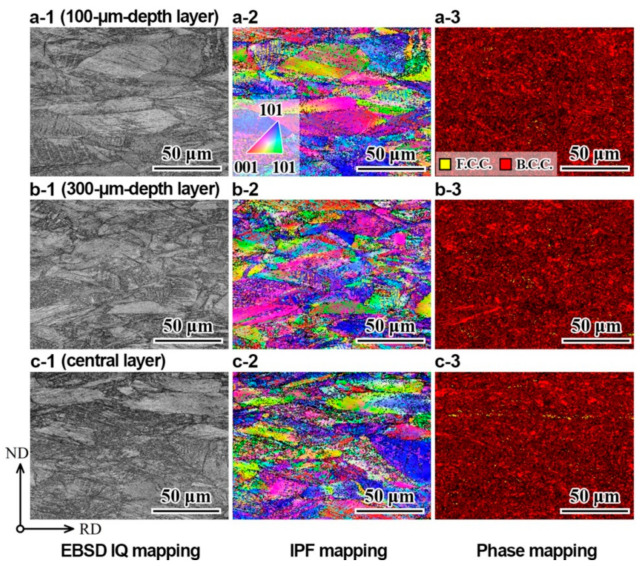
EBSD images of the GS2 sample after tensile testing at cryogenic temperature: (**a1**–**a3**) IQ, IPF and phase mappings for the layer with a depth of 100 µm; (**b1**–**b3**) IQ, IPF and phase mappings for the layer with a depth of 300 µm; (**c1**–**c3**) IQ, IPF and phase mappings for the central layer.

The microstructures after tensile deformation at both room and cryogenic temperatures for the GS2 sample were characterized by EBSD and are displayed in Figure 6 and Figure 7, respectively. The IQ, IPF and phase mappings of the GS2 sample after tensile testing at room temperature for the layer with a depth of 100 µm and the central layer are displayed in Figure 6(a1–a3) and Figure 6(b1–b3), respectively. At all depths, the dislocation density is higher as compared to that before testing. At all depths, high density of dislocations, dislocation walls and cells are formed in the grain interior. The densities of dislocations, dislocation walls and cells are observed to be higher at the surface layer as compared to the central layer. Moreover, multiple deformation twins are observed to form in the austenite grain interior, as indicated by the point-to-point misorientation along the marked line in the inset of Figure 6(a2) (typical misorientation angle of 600 for twinning). It is generally thought that multiple twinning can induce higher strength and stronger strain hardening than dislocation behaviors or single DTs [16,27,29]. The multiple twinning network not only can present more barriers for dislocation sliding to contribute to the higher strength, and the network also can provide adequate pathways for easy gliding and cross-slip of dislocations to accommodate significant plastic deformation for stronger strain hardening [27,29].

Previous results [38,62] have shown that there is no martensitic transformation for the CG 304 ss during tensile deformation at room temperature. However, significant martensitic transformation has been observed at all depths for the GS2 sample after tensile testing at room temperature (Figure 6a(3),b(3)), which might be one of the origins for the better synergy of strength and ductility in the gradient structures. It is also interesting to note that the martensitic transformation is more obvious at the central layer as compared to the layer with a depth of 100 µm, which might be attributed to higher density of the RXGs with small grain size (UFGs) at the surface layer. It is generally thought that smaller grain size can inhibit the martensitic transformation [54,55]. It is observed that the martensite phase is formed at the intersection regions of deformation twins from different slip systems or the interfaces for dislocation walls and cells. These regions generally can induce high stress to promote martensitic transformation.

The IQ, IPF and phase mappings of the GS2 sample after tensile testing at cryogenic temperature for the layer with a depth of 100 µm, the layer with a depth of 300 µm and the central layer are displayed in Figure 7a(1–3), Figure 7b(1–3) and Figure 7c(1–3), respectively.

**Figure 8:**
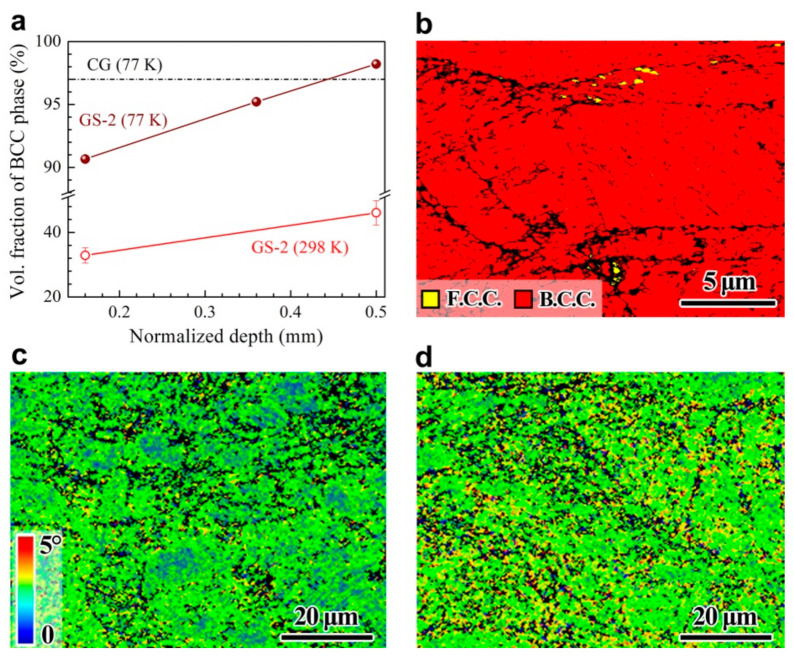
(**a**) Volume fraction of martensitic transformation as a function of normalized depth for the GS2 sample after testing at both 298 and 77 K. (**b**) Phase image for the CG sample after tensile testing at 77 K. (**c**,**d**) KAM mappings after tensile testing at 77 K for the CG sample and the central layer of the GS2 sample, respectively.

The volume fraction of martensitic transformation is plotted as a function of normalized depth for the GS2 sample during testing at both room and cryogenic temperatures in Figure 8a, in which the volume fraction of martensitic transformation for the CG sample during testing at cryogenic temperature is marked by a dash line. Compared to testing at room temperature, the martensitic transformation is more obvious after testing at cryogenic temperature for all depths, resulting in stronger strain hardening for the gradient structures. The amount of martensitic transformation at the central layer of the GS2 sample is observed to be even higher than that for the CG sample at cryogenic temperature. This might be one of the origins for the simultaneous improvement strength and ductility by the gradient structures at cryogenic temperature. Moreover, the phase and KAM mappings of the CG sample after tensile testing at cryogenic temperature are shown in Figure 8b,c, respectively.

Furthermore, the amount of GNDs is also observed to be much higher at the central layer of the GS sample (Figure 8d), as compared to the CG sample (Figure 8c) after tensile testing at cryogenic temperature. The GND densities of the CG sample and the central layer of the GS2 sample after tensile testing at cryogenic temperature are 1.2 × 10^17^ m^−2^ and 1.7 × 10^17^ m^−2^ (based on Figure 8c,d and the aforementioned equation, $\rho_{GND}=2\theta /lb$), respectively. This is another origin for the simultaneous improvement strength and ductility by the gradient structures at cryogenic temperature.

## 4. Conclusions

In the present study, gradient structures were produced via the SMAT technique and the subsequent short-time annealing in the 304 ss. Then, the tensile properties and the corresponding deformation mechanisms of the graded 304 ss at both room and cryogenic temperatures were investigated and compared with those of the CGed 304 ss. The findings can be summarized as follows:

(1). Compared to the homogeneous CG sample, excellent synergy of strength and ductility was obtained by the gradient structures at room temperature. Moreover, simultaneous improvement of the yield strength and the uniform elongation was induced by the gradient structures at cryogenic temperature, as compared to the homogeneous CG sample.
(2). The HDI hardening was found to play a more important role in the gradient structures as compared to the homogeneous CG sample at both room and cryogenic temperatures, resulting in better tensile properties in the gradient structures. The HDI hardening was also found to be much more obvious at cryogenic temperature as compared to that at room temperature.
(3). In the gradient structures, the central layer provides stronger strain hardening during tensile deformation at both room and cryogenic temperatures, as compared to the surface layer. The volume fraction of martensitic transformation was found to increase along the depth from the surface layer to the central layer during tensile deformation at both room and cryogenic temperatures for the gradient structures. The cryogenic deformation was found to induce more volume fraction of martensitic transformation at each depth in the gradient structures as compared to the room temperature deformation, resulting in a much stronger strain hardening at cryogenic temperature.
(4). The dislocation density and the amount of martensitic transformation are observed to be higher at the central layer of the gradient structures as compared to the CG sample, which are the origins for the simultaneous improvement strength and ductility by the gradient structures at the cryogenic temperature. The present findings should provide insights for tailoring heterogeneous microstructures to achieve excellent synergy of strength and ductility/toughness at both room and cryogenic temperatures in metals and alloys.

## Data Availability

The data presented in this study are available on reasonable requests from the corresponding author.
